# Genetic mutations in *Plasmodium falciparum* and *Plasmodium vivax* dihydrofolate reductase (DHFR) and dihydropteroate synthase (DHPS) in Vanuatu and Solomon Islands prior to the introduction of artemisinin combination therapy

**DOI:** 10.1186/1475-2875-13-402

**Published:** 2014-10-14

**Authors:** Karryn J Gresty, Karen-Ann Gray, Albino Bobogare, Lyndes Wini, George Taleo, Jeffrey Hii, Qin Cheng, Norman C Waters

**Affiliations:** Australian Army Malaria Institute, Enoggera, Brisbane, Queensland Australia; QIMR Berghofer Medical Research Institute, Brisbane, Queensland Australia; Malaria and Vector Borne Diseases Control Programme, Ministry of Health, Honiara, Solomon Islands; Vector Borne Disease Control Program, Ministry of Health, Port Vila, Vanuatu; School of Public Health, Tropical Medicine and Rehabilitation Sciences, James Cook University, Townsville, Queensland Australia; Walter Reed Army Institute of Research, Malaria Vaccine Branch, Military Malaria Research Program, Silver Spring, Maryland USA

**Keywords:** *Plasmodium falciparum*, *Plasmodium vivax*, Surveillance, Molecular markers, Sulphadoxine-pyrimethamine, *dhfr*, *dhps*, Vanuatu, Solomon Islands

## Abstract

**Background:**

*Plasmodium falciparum* and *Plasmodium vivax* are endemic in Vanuatu and the Solomon Islands. While both countries have introduced artemether-lumefantrine (AL) as first-line therapy for both *P. falciparum* and *P. vivax* since 2008, chloroquine and sulphadoxine-pyrimethamine (SP) were used as first-line therapy for many years prior to the introduction of AL. Limited data are available on the extent of SP resistance at the time of policy change.

**Methods:**

Blood spots were obtained from epidemiological surveys conducted on Tanna Island, Tafea Province, Vanuatu and Temotu Province, Solomon Islands in 2008. Additional samples from Malaita Province, Solomon Islands were collected as part of an AL therapeutic efficacy study conducted in 2008. *Plasmodium vivax* and *P. falciparum dhfr* and *dhps* genes were sequenced to detect nucleotide polymorphisms.

**Results:**

All *P. falciparum* samples analysed (n =114) possessed a double mutant *pfdhfr* allele (C59**R**/S108**N)***.* Additionally, mutation A437**G** in *pfhdps* was detected in a small number of samples 2/13, 1/17 and 3/26 from Tanna Island, Vanuatu and Temotu and Malaita Provinces Solomon Islands respectively. Mutations were also common in *pvdhfr* from Tanna Island, Vanuatu, where 33/51 parasites carried the double amino acid substitution S58**R**/S117**N**, while in Temotu and Malaita Provinces, Solomon Islands 32/40 and 39/46 isolates carried the quadruple amino acid substitution F57**L**/S58**R**/T61**M**/S117**T** in DHFR respectively. No mutations in *pvdhps* (n =108) were detected in these three island groups.

**Conclusion:**

Prior to the introduction of AL, there was a moderate level of SP resistance in the *P. falciparum* population that may cause SP treatment failure in young children. Of the *P. vivax* isolates, a majority of Solomon Islands isolates carried quadruple mutant *pvdhfr* alleles while a majority of Vanuatu isolates carried double mutant *pvdhfr* alleles. This suggests a higher level of SP resistance in the *P. vivax* population in Solomon Islands compared to the sympatric *P. falciparum* population and there is a higher level of SP resistance in *P. vivax* parasites from Solomon Islands than Vanuatu. This study demonstrates that the change of treatment policy in these countries from SP to ACT was timely. The information also provides a baseline for future monitoring.

## Background

Both *Plasmodium falciparum* and *Plasmodium vivax* are endemic in Vanuatu (except for the islands of Aneityum and Futuna which are malaria free) and the Solomon Islands. Malaria transmission throughout Vanuatu and the Solomon Islands occurs year round with varying degrees of seasonal intensity. In Vanuatu, transmission peaks from December to April, while in the Solomon Islands there are two transmission peaks, April to September and November to February. Over the past decade malaria incidence rates have declined, and today, both countries are intensifying malaria control country wide and progressing towards malaria elimination in targeted provinces.

The success of malaria control and elimination programs in these countries relies on effective treatment of malaria cases and asymptomatic carriers. Historically, both countries used chloroquine (CQ) for treatment of uncomplicated *P. falciparum* malaria. Chloroquine-resistance (CQR) in *P. falciparum* was first reported in the early 1980s [1] and subsequent loss of CQ efficacy forced a treatment policy change in the Solomon Islands and Vanuatu [2]. In 1994, the Vanuatu Ministry of Health (MOH) introduced a combination therapy of CQ and sulphadoxine-pyrimethamine (SP) for treatment of uncomplicated *P. falciparum* infection, while CQ remained the prescribed treatment for *P. vivax* infections [2]. Similarly, in the Solomon Islands throughout the 1990s the combination of CQ + SP was used as second-line treatment and later from 2001 to 2007, as first-line treatment [2].

In 2008–2009, following WHO recommendation, Vanuatu and the Solomon Islands introduced artemether-lumefantrine (AL) as first-line treatment for uncomplicated falciparum malaria, and AL plus primaquine for treatment of malaria caused by *P. vivax* [3]. These are two of the few countries where AL is used to treat both vivax and falciparum malaria, however there was limited information on the extent of parasite resistance to CQ and SP at the time of AL implementation.

SP is one of the most extensively used anti-malarials throughout the world; the SP drug combination primarily targets dihydrofolate reductase (DHFR) and dihydropteroate synthase (DHPS), critical enzymes in the folate biosynthetic pathway of the malarial parasite, thereby killing the parasite. However, parasites rapidly develop resistance to SP through single nucleotide polymorphisms (SNPs) in the genes encoding DHFR and DHPS enzymes [4–6]. Drug resistant mutations occurring mainly in five amino acid residues situated close to the active site on the *P. falciparum* DHFR (A16**V**, N51**I**, C59**R**, S108**N**/**T** and I164**L**) and in four amino acid positions (F57**I**/**L**, S58**R*,*** T61**M** and S117**T**/**N**) in *P. vivax* DHFR have been widely reported [7–13]. Combinations of these mutations correlate with varying levels of SP resistance in parasites. Mutations in *P. falciparum* DHPS (S436**A**, A437**G*,*** K540**E**, A581**G** and A613**S**/**T*)*** confer resistance to sulphadoxine [6]. Analogous mutations in the *P. vivax* DHPS gene have also been determined (A383**G**, A553**G** and V585) [14, 15] *.*

The ability to monitor for mutations in the *P. falciparum* and *P. vivax dhfr* and *dhps* genes provides a valuable method of epidemiological surveillance of SP resistance. For instance, areas experiencing higher levels of SP pressure tend to exhibit increased prevalence of *pvdhfr* mutant alleles while those areas under little or no SP pressure are more likely to maintain the wild-type *pvdhfr* genotype [10, 11]. Since the introduction of SP in early 1990s, two surveys have been conducted in Guadalcanal, Solomon Islands (1995–1996 [13], 2004–2005 [16]) and Malo Island, Vanuatu (1996–1998 [13], 2005 [17]). However, due to fragmented land masses in both countries, parasite population and drug resistance profiles could be very different between different islands.

This paper describes prevalence of *P. falciparum* and *P. vivax* parasites resistant to SP in three areas of Vanuatu and Solomon Islands prior to the implementation of AL. The data and results reported here provide excellent baseline information on the status of SP resistant mutations in parasite populations prior to changes in treatment guidelines. The information also enables investigations into evolution of SP resistance in *P. falciparum* and *P. vivax* populations following a parallel change of treatment policy in areas where both *Plasmodium* species co-exist.

## Methods

### Study areas and sample collection

Three sets of blood samples were used in this study. Two sets were obtained from epidemiological surveys conducted (2008) during the wet seasons in Tafea Province, Vanuatu and Temotu Province, Solomon Islands, the geographic locations and information regarding the demographics of the populations surveyed and consent process have been published previously [18]. In brief, a school-based, mass blood survey of children (2–12 years) was performed on Tanna Island, Tafea Province which is the most southern Province of Vanuatu. In this study speciation by PCR identified 42 samples positive for *P. falciparum* infection, 92 for *P. vivax* infection and 10 for mixed *P. falciparum/P. vivax* infections. Similarly, a village-based mass blood survey encompassing all ages was conducted on Temotu Province, the most southern Province in the Solomon Islands which is made up of five main island groups: Santa Cruz, Reef Islands, Duff Islands, Utupua Island and Vanikoro Island. In this study PCR identified 118 *P. falciparum* infections, 162 *P. vivax* infections and 39 mixed *P. falciparum/P. vivax* infections. The third set of samples was obtained from febrile patients attending Auki town clinic and Kilu’Ufi hospital, Malaita Province, Solomon Islands through collaboration with the World Health Organization (WHO) and Ministry of Health, Solomon Islands. These samples were collected as part of an artemether-lumefantrine (AL, Coartem™, Novartis) efficacy study conducted within the Solomon Islands planned and executed by the WHO and MOH Solomon Islands. Malaita Province lies approximately 9°S 161°E and is the second largest and most densely populated Province of the Solomon Islands. In this study PCR identified 68 *P. falciparum* infections, 31 *P. vivax* infections and 37 mixed *P. falciparum/P. vivax* infections. Across all study sites blood samples were collected by finger prick and a blood spot (20–30 μL) was air-dried onto filter paper (Whatman No. 3), sealed in individual plastic bags containing desiccant and stored at room temperature until further processing.

### Extraction of parasite DNA

Genomic DNA was extracted from blood samples that were *P. falciparum* or *P. vivax* positive by microscopy using QIAGEN QIAamp DNA Mini Kits and a QIAcube robot (QIAGEN, Crawley, U.K.). The manufacturer’s protocol (QIAamp DNA Mini and Blood Mini Handbook 2E) was followed with the modifications that only one 5 mm circle was extracted and each sample was eluted using 100 μL of AE buffer.

### Confirmation of *Plasmodium spp*

*Plasmodium* speciation was performed by multiplex PCRs [19] (*P. falciparum, P. vivax, Plasmodium ovale* or *Plasmodium malariae*) followed by a confirmatory single species PCR, as previously described [18, 20].

### Amplification of *dhfr*and *dhps*genes

Approximately 30% of PCR confirmed *Plasmodium* positive samples were selected at random for DNA sequence analysis. These samples were being used for several different studies, we were therefore limited in the number of samples we were able to analyse. DNA fragments of the *dhfr* and *dhps* genes of *P. falciparum* and *P. vivax* encompassing sites of known mutations were amplified by nested PCR as described previously [6, 14, 21, 22]. PCR products were purified using ExoSAP-IT^R^ (USB, Cleveland, OH) to remove excess primers and nucleotides according to the manufacturer’s protocol and then sequenced. Sequencing was performed using Big Dye Terminator (v.3.1) on an automated DNA sequencer, ABI 3100 system, at the QIMR Berghofer Medical Research Institute Scientific Services Analytical Facility. In cases where PCR products contained multiple bands, a single band of expected size was excised from an agarose gel and purified using a NucleoSpin^R^ extraction kit (Macherey-Nagel) and then sequenced. Sequence polymorphisms were determined by alignment.

## Results

### Mutations in *P. falciparum*DHFR and DHPS

The *dhfr* and *dhps* genes of *P. falciparum* were amplified and sequenced from randomly selected samples from all three island groups. The number of samples sequenced for these genes are detailed in Table 1. Sequencing analysis of *pfdhfr* revealed all isolates (n = 114) from all three island groups exhibited amino acid substitutions C59**R** and S108**N** having an allelic type AC**RN**I. Analysis of *pfdhps* revealed that 11/13, 16/17 and 23/26 samples were of wild type allele SAKAA, from Tanna Island, Vanuatu and Temotu and Malaita Provinces Solomon Islands, respectively. In addition one sample from Tanna Island, Vanuatu and three samples from Malaita Province, Solomon Islands possessed a single A437**G** substitution while one sample from Port Resolution on Tanna Island, Vanuatu was identified with a double substitution A437**G** + A581**G**. Furthermore, one sample from Utupua Island, Temotu Province, Solomon Islands carried a rare but widespread S436**F** mutation in conjunction with A437**G** and A613**S** substitutions resulting in a triple mutant allele **FG**KA**S** (Table 1).

**Table 1:** **Amino acid substitutions (bold) and prevalence in** ***P. falciparum*** **DHFR and DHPS from Vanuatu and Solomon Islands**

PfDHFR								
Amino acid position		n	Frequency	16	51	59	108	164
Wild type				A	N	C	S	I
Known substitutions in the region				**V**	**I**	**R**	**N/T**	**L**
Country	Island/province							
Vanuatu	Tanna Is.	22	22	A	C	**R**	**N**	I
Solomon Is	Temotu Prov.	34	34	A	C	**R**	**N**	I
	Malaita Prov.	58	58	A	C	**R**	**N**	I
Pf DHPS								
Amino acid position		n	Frequency	436	437	540	581	613
Wild type				S	A	K	A	A
Known substitutions in the region				**A/F**	**G**	**E**	**G**	**S/T**
Country	Island/province							
Vanuatu	Tanna Is.	13	11	S	A	K	A	A
			1	S	**G**	K	A	A
			1	S	**G**	K	**G**	A
Solomon Is	Temotu Prov.	17	16	S	A	K	A	A
			1	**F**	**G**	K	A	**S**
	Malaita Prov.	26	23	S	A	K	A	A
			3	S	**G**	K	A	A

### Mutations in *Plasmodium vivax*DHFR and DHPS

On Tanna Island, Vanuatu, 33/51 *P. vivax* isolates carried a double mutant *pvdhfr* allele with substitutions S58**R**/S117**N**. A further eleven isolates were triple mutant alleles P**M**ST**TF**, containing a novel F57**M** substitution, while the remaining seven isolates were double mutant alleles P**L**STS**F**. In Temotu Province, Solomon Islands there was a high incidence of samples containing mixed strains; these were excluded from further analysis. The dominant allele occurring in 32/40 samples was the *pvdhfr* quadruple mutant allele P**LRMT**I. Of the remaining isolates, four were single mutant alleles S117**N**, three were triple mutant alleles P**LR**T**T**I and one was a triple mutant allele P**LR**TS**M**. In contrast to *pvdhfr*, all *P. vivax* isolates (n = 108) analysed from all three regions possessed only a wild type *pvdhps* allele SAKAV in conjunction with one or more mutations in *pvdhfr* (Table 2).

**Table 2:** **Amino acid substitutions (bold) and prevalence in** ***P. vivax*** **DHFR and DHPS from Vanuatu and Solomon Islands**

PvDHFR									
Amino acid position		n	Frequency	33	57	58	61	117	173
Wild type				P	F	S	T	S	I
Known substitutions in the region				**L**	**I/L**	**R**	**N**	**T/N**	**L**
Country	Island/province								
Vanuatu	Tanna Is.	51	33	P	F	**R**	T	**N**	I
			11	P	**M**	S	T	**T**	**F**
			7	P	**L**	S	T	S	**F**
Solomon Is	Temotu Prov.	40	32	P	**L**	**R**	**M**	**T**	I
			4	P	F	S	T	**N**	I
			3	P	**L**	**R**	T	**T**	I
			1	P	**L**	**R**	T	S	**M**
	Malaita Prov.	46	39	P	**L**	**R**	**M**	**T**	I
			3	P	**L**	**R**	T	S	**M**
			2	P	F	**R**_AGA_	T	**N**	I
			1	P	**L**	**R**	T	**N**	I
			1	P	F	**R**_AGA_	T	**T**	I
PvDHPS									
Amino acid position		n	Frequency	382	383	512	553	585	
Wild type				S	A	K	A	V	
Known substitutions in the region					**G**		**G**		
Country	Island/province								
Vanuatu	Tanna Is.	38	38	S	A	K	A	V	
Solomon Is	Temotu Prov.	22	22	S	A	K	A	V	
	Malaita Prov.	48	48	S	A	K	A	V	

Similarly, in Malaita Province, Solomon Islands, 39/46 isolates carried a *pvdhfr* quadruple mutant allele P**LRMT**I. A further three samples were triple mutant alleles P**LR**TS**M** carrying a novel I173**M** mutation, with another two samples identified as double mutant alleles PF**R**_AGA_T**N**I. The remaining allelic types consisted of single isolates P**LR**T**N**I and PF**R**_AGA_T**T**I. Interestingly, in Malaita Province, Solomon Islands, three samples possessed a S58**R** substitution where the arginine residue was coded by AGA, whereas in Temotu Province and Tanna Island, Vanuatu the arginine was solely coded by AGG (Table 2).

## Discussion

Vanuatu and the Solomon Islands are among the few countries where ACT is used for treatment of both *P. falciparum* and *P. vivax* malaria. Understanding the parasite drug resistance profile at the time of ACT introduction allows for the detection of changes in future parasite populations. Although it is not clear whether parasites carrying mutant *dhfr* and *dhps* alleles have a selective advantage or disadvantage under AL pressure, the aim of this paper was to determine the base line polymorphisms of *pfdhfr, pfhdps, pvdfr and pvdhps* at the time of AL introduction in three island groups not previously surveyed.

Results of this study indicated that 100% of *P. falciparum* and *P. vivax* isolates from three island groups in Vanuatu and the Solomon Islands had point mutations in the gene coding for DHFR that are associated with antifolate resistance. In contrast, an average of 88.6% of *P. falciparum* isolates and 100% of *P. vivax* isolates possessed a wild type *dhps* gene. The differences in the prevalence of mutations between DHFR and DHPS supports the asymmetric selection pattern previously reported [7] to occur in both species in these areas.

A double mutant *pfdhfr* allele, C59**R**/S108**N,** was detected in 100% of *P. falciparum* isolates tested from all three island groups. This pattern and fixation is similar to that reported for parasites collected on different islands to this study in the mid1990s and in 2005 [13, 16, 17]. Based on earlier studies, point mutation S108**N** alone is sufficient to confer resistance to pyrimethamine [5, 23–25], the addition of a C59**R** mutation in conjunction with S108**N** would result in a sizeable increase in the levels of resistance to pyrimethamine [5, 23–25]. Although this double mutant allele does not confer SP resistance levels as high as the quadruple mutant alleles [26], in some studies it has been implicated in treatment failures in young children [27, 28], while other studies indicate minimal SP treatment failures [29, 30]. The fixation of double mutant *pfdhfr* alleles in *P. falciparum* populations in Vanuatu and Solomon Islands indicates that children infected with *P. falciparum* could potentially experience treatment failure if SP usage was continued.

Previously published findings at different island locations in Vanuatu and Solomon Islands reported only wild type *pfdhps* alleles [13, 16, 17]. In this study, although a majority of the *P. falciparum* isolates (50/56) from all three island groups had a wild type *pfdhps*, a small number of samples (6) possessed an A437**G** mutation. The *pfdhps* A437**G** mutation is often observed in regions where SP has been widely used and its presence alone, although more commonly when combined with mutation K540**E** and a triple DHFR mutation, is predictive of early SP treatment failure [7, 28, 31]. In this study, no mutations were detected at amino acid position 540 of *pfdhps* in any of the samples investigated. This is not unexpected since earlier studies have indicated that the presence of K540**E** is often associated in the quintuple haplotype including three mutations in *pfdhfr* and two mutations in *pfdhps* [31]. Interestingly, one sample from Port Resolution on Tanna Island, Vanuatu was identified as carrying a double mutant *pfdhps* allele A437**G** and A581**G**. This suggests that parasite populations were exposed to SP pressure since A581**G** is reported to increase in frequency with extended SP usage [32]. It is also possible that this parasite isolate was imported from other areas because the village is located in a busy port serving inter-island sea travel.

The occurrence of double, triple and quadruple mutations in *pvdhfr* were observed in isolates from Tanna Island, Vanuatu as well as Temotu and Malaita Provinces, Solomon Islands. In Vanuatu the dominant allele occurring in 64% of isolates was the double mutant allele PNF**R**T**N**I, whereas in Malaita and Temotu Provinces, Solomon Islands, the dominant allele was the quadruple mutant allele PN**LRMT**I occurring in 85% and 80% of isolates, respectively. Amino acid codon 117 of *pvdhfr* has been reported to mutate to either an asparagine (N) or threonine (T). While S117**N** has been associated with reduced *in vitro* susceptibility to pyrimethamine it has not been reported to associate with the highly resistant quadruple mutant alleles, and has therefore been hypothesized as a “dead end” in resistance progression. In contrast, the S117**T** mutation has been associated with quadruple mutant alleles and clinical resistance to SP [22, 33]. On the other hand, the *pvdhfr* double mutants S58**R** + S117**N** are usually associated with delayed parasite clearance after SP treatment, not failure. This indicates that *P. vivax* isolates in Solomon Islands may have been under more extensive selection pressure than those in Tanna Island, Vanuatu. Mass drug administration of chloroquine and primaquine was one of the key preventive strategies used by the Malaria Eradication Programme in Solomon Islands from 1974 [34] to the early 1990s.

Interestingly only wild type *pvdhps* alleles were detected in isolates from all three island groups. All *P. vivax* isolates had V585 that was previously reported to play a major role in the innate resistance of this parasite to sulphadoxine [14].

## Conclusion and implications

This study provides baseline data on the extent of SP drug resistance in *P. vivax* and *P. falciparum* parasites in these three island groups prior to the introduction of ACT. In summary, 100% of *P. falciparum* parasites carried a double mutant *pfdhfr* allele with a small proportion of these also carrying a single or double *dhps* mutation. This indicates a moderate level of SP resistance in the *P. falciparum* populations that may cause SP treatment failure in young children. Similarly 100% of *P. vivax* isolates carried mutant *pvdhfr* alleles: a dominant double mutant allele in Vanuatu and a dominant quadruple mutant allele in Solomon Islands. It is speculated that *P. vivax* has developed a higher level of SP resistance in Solomon Islands compared to the sympatric *P. falciparum* population, and there is a higher level of SP resistance in *P. vivax* parasites from Solomon Islands than Vanuatu. This study demonstrates that the change of treatment policy from CQ + SP to ACT was timely.

## References

[1] Bowden DK, Bastien P, Douglas FP, Muir JW, Tambisari E (1982). Chloroquine-resistant *Plasmodium falciparum* malaria in Vanuatu. Med J Aust.

[2] WHO (2005). Review of the Malaria Drug Efficacy Situation in 10 Countries of the WHO Western Pacific Region 1987–2003.

[3] WHO (2013). World Malaria Report 2013.

[4] Peterson DS, Walliker D, Wellems TE (1988). Evidence that a point mutation in dihydrofolate reductase-thymidylate synthase confers resistance to pyrimethamine in falciparum malaria. Proc Natl Acad Sci US A.

[5] Cowman AF, Morry MJ, Biggs BA, Cross GA, Foote SJ (1988). Amino acid changes linked to pyrimethamine resistance in the dihydrofolate reductase-thymidylate synthase gene of *Plasmodium falciparum*. Proc Natl Acad Sci U S A.

[6] Triglia T, Menting JG, Wilson C, Cowman AF (1997). Mutations in dihydropteroate synthase are responsible for sulfone and sulfonamide resistance in *Plasmodium falciparum*. Proc Natl Acad Sci U S A.

[7] Sibley CH, Hyde JE, Sims PF, Plowe CV, Kublin JG, Mberu EK, Cowman AF, Winstanley PA, Watkins WM, Nzila AM (2001). Pyrimethamine-sulfadoxine resistance in *Plasmodium falciparum*: what next?. Trends Parasitol.

[8] Roper C, Pearce R, Nair S, Sharp B, Nosten F, Anderson T (2004). Intercontinental spread of pyrimethamine-resistant malaria. Science.

[9] Nair S, Williams JT, Brockman A, Paiphun L, Mayxay M, Newton PN, Guthmann JP, Smithuis FM, Hien TT, White NJ, Nosten F, Anderson TJ (2003). A selective sweep driven by pyrimethamine treatment in southeast asian malaria parasites. Mol Biol Evol.

[10] Imwong M, Pukrittakayamee S, Looareesuwan S, Pasvol G, Poirreiz J, White NJ, Snounou G (2001). Association of genetic mutations in *Plasmodium vivax* dhfr with resistance to sulfadoxine-pyrimethamine: geographical and clinical correlates. Antimicrob Agents Chemother.

[11] Imwong M, Pukrittayakamee S, Renia L, Letourneur F, Charlieu JP, Leartsakulpanich U, Looareesuwan S, White NJ, Snounou G (2003). Novel point mutations in the dihydrofolate reductase gene of *Plasmodium vivax*: evidence for sequential selection by drug pressure. Antimicrob Agents Chemother.

[12] Hawkins VN, Auliff A, Prajapati SK, Rungsihirunrat K, Hapuarachchi HC, Maestre A, O'Neil MT, Cheng Q, Joshi H, Na-Bangchang K, Sibley CH (2008). Multiple origins of resistance-conferring mutations in *Plasmodium vivax* dihydrofolate reductase. Malar J.

[13] Mita T, Tanabe K, Takahashi N, Tsukahara T, Eto H, Dysoley L, Ohmae H, Kita K, Krudsood S, Looareesuwan S, Kaneko A, Bjorkman A, Kobayakawa T (2007). Independent evolution of pyrimethamine resistance in *Plasmodium falciparum* isolates in Melanesia. Antimicrob Agents Chemother.

[14] Korsinczky M, Fischer K, Chen N, Baker J, Rieckmann K, Cheng Q (2004). Sulfadoxine resistance in *Plasmodium vivax* is associated with a specific amino acid in dihydropteroate synthase at the putative sulfadoxine-binding site. Antimicrob Agents Chemother.

[15] Imwong M, Pukrittayakamee S, Cheng Q, Moore C, Looareesuwan S, Snounou G, White NJ, Day NP (2005). Limited polymorphism in the dihydropteroate synthetase gene (dhps) of *Plasmodium vivax* isolates from Thailand. Antimicrob Agents Chemother.

[16] Ballif M, Hii J, Marfurt J, Crameri A, Fafale A, Felger I, Beck HP, Genton B (2010). Monitoring of malaria parasite resistance to chloroquine and sulphadoxine-pyrimethamine in the Solomon Islands by DNA microarray technology. Malar J.

[17] Kinzer MH, Chand K, Basri H, Lederman ER, Susanti AI, Elyazar I, Taleo G, Rogers WO, Bangs MJ, Maguire JD (2010). Active case detection, treatment of *falciparum* malaria with combined chloroquine and sulphadoxine/pyrimethamine and *vivax* malaria with chloroquine and molecular markers of anti-malarial resistance in the Republic of Vanuatu. Malar J.

[18] The Pacific Malaria Initiative Survey Group (2010). Malaria on isolated Melanesian islands prior to the initiation of malaria elimination activities. Malar J.

[19] Padley D, Moody AH, Chiodini PL, Saldanha J (2003). Use of a rapid, single-round, multiplex PCR to detect malarial parasites and identify the species present. Ann Trop Med Parasitol.

[20] Harris I, Sharrock WW, Bain LM, Gray KA, Bobogare A, Boaz L, Lilley K, Krause D, Vallely A, Johnson ML, Gatton ML, Shanks GD, Cheng Q (2010). A large proportion of asymptomatic *Plasmodium* infections with low and sub-microscopic parasite densities in the low transmission setting of Temotu Province, Solomon Islands: challenges for malaria diagnostics in an elimination setting. Malar J.

[21] Basco LK, Eldin-de PP, Wilson CM, Le-Bras J, Mazabraud A (1995). Point mutations in the dihydrofolate reductase-thymidylate synthase gene and pyrimethamine and cycloguanil resistance in *Plasmodium falciparum*. Mol Biochem Parasitol.

[22] Tjitra E, Baker J, Suprianto S, Cheng Q, Anstey NM (2002). Therapeutic efficacies of artesunate-sulfadoxine-pyrimethamine and chloroquine-sulfadoxine-pyrimethamine in vivax malaria pilot studies: relationship to *Plasmodium vivax* dhfr mutations. Antimicrob Agents Chemother.

[23] Wang P, Lee CS, Bayoumi R, Djimde A, Doumbo O, Swedberg G, Dao LD, Mshinda H, Tanner M, Watkins WM, Sims PF, Hyde JE (1997). Resistance to antifolates in *Plasmodium falciparum* monitored by sequence analysis of dihydropteroate synthetase and dihydrofolate reductase alleles in a large number of field samples of diverse origins. Mol Biochem Parasitol.

[24] Sirawaraporn W, Sirawaraporn R, Cowman AF, Yuthavong Y, Santi DV (1990). Heterologous expression of active thymidylate synthase-dihydrofolate reductase from *Plasmodium falciparum*. Biochemistry.

[25] Wooden JM, Hartwell LH, Vasquez B, Sibley CH (1997). Analysis in yeast of antimalaria drugs that target the dihydrofolate reductase of *Plasmodium falciparum*. Mol Biochem Parasitol.

[26] Hankins EG, Warhurst DC, Sibley CH (2001). Novel alleles of the *Plasmodium falciparum* dhfr highly resistant to pyrimethamine and chlorcycloguanil, but not WR99210. Mol Biochem Parasitol.

[27] Talisuna AO, Nalunkuma-Kazibwe A, Langi P, Mutabingwa TK, Watkins WW, Van Marck E, Egwang TG, D’Alessandro U (2004). Two mutations in dihydrofolate reductase combined with one in the dihydropteroate synthase gene predict sulphadoxine-pyrimethamine parasitological failure in Ugandan children with uncomplicated *falciparum* malaria. Infect Genet Evol.

[28] Kyabayinze D, Cattamanchi A, Kamya MR, Rosenthal PJ, Dorsey G (2003). Validation of a simplified method for using molecular markers to predict sulfadoxine-pyrimethamine treatment failure in African children with falciparum malaria. Am J Trop Med Hyg.

[29] Dunyo S, Ord R, Hallett R, Jawara M, Walraven G, Mesa E, Coleman R, Sowe M, Alexander N, Targett GAT, Pinder M, Sutherland CJ (2006). Randomised trial of chloroquine/sulphadoxine-pyrimethamine in Gambian Children with malaria: impact against multidrug-resistant *P. falciparum*. PLoS Clin Trials.

[30] Hallett RL, Dunyo S, Ord R, Jawara M, Pinder M, Randall A, Alloueche A, Walraven G, Targett GAT, Alexander N, Sutherland CJ (2006). Chloroquine/sulphadoxine-pyrimethamine for Gambian children with malaria: transmission to mosquitoes of multidrug-resistant *Plasmodium falciparum*. PLoS Clin Trials.

[31] Kublin JG, Dzinjalamala FK, Kamwendo DD, Malkin EM, Cortese JF, Martino LM, Mukadam RA, Rogerson SJ, Lescano AG, Molyneux ME, Winstanley PA, Chimpeni P, Taylor TE, Plowe CV (2002). Molecular markers for failure of sulfadoxine-pyrimethamine and chlorproguanil-dapsone treatment of *Plasmodium falciparum* malaria. J Infect Dis.

[32] Alifrangis M, Lusingu JP, Mmbando B, Dalgaard MB, Vestergaard LS, Ishengoma D, Khalil IF, Theander TG, Lemnge MM, Bygbjerg IC (2009). Five-year surveillance of molecular markers of *Plasmodium falciparum* antimalarial drug resistance in Korogwe District, Tanzania: accumulation of the 581G mutation in the *P. falciparum* dihydropteroate synthase gene. Am J Trop Med Hyg.

[33] Hastings MD, Porter KM, Maguire JD, Susanti I, Kania W, Bangs MJ, Hopkins SC, Baird JK (2004). Dihydrofolate reductase mutations in *Plasmodium vivax* from Indonesia and therapeutic response to sulfadoxine slus pyrimethamine. J Infect Dis.

[34] Saint-Yves IF (1975). Results of two Treatment Schedules for Plasmodium Vivax Infections in the Solomon Islands Malaria Eradication Programme.

